# In Vitro and Vivo Identification, Metabolism and Action of Xenoestrogens: An Overview

**DOI:** 10.3390/ijms22084013

**Published:** 2021-04-13

**Authors:** Li-Hsuan Wang, Li-Ru Chen, Kuo-Hu Chen

**Affiliations:** 1 Department of Obstetrics and Gynecology, Taipei Tzu-Chi Hospital, The Buddhist Tzu-Chi Medical Foundation, Taipei 231, Taiwan; 100311023@gms.tcu.edu.tw; 2 Department of Physical Medicine and Rehabilitation, Mackay Memorial Hospital, Taipei 10049, Taiwan; gracealex168@gmail.com; 3 Department of Mechanical Engineering, National Yang-Ming Chiao-Tung University, Hsinchu 30010, Taiwan; 4 School of Medicine, Tzu-Chi University, Hualien 970, Taiwan

**Keywords:** xenoestrogen, bisphenol, polychlorinated biphenyls, phytoestrogen

## Abstract

Xenoestrogens (XEs) are substances that imitate endogenous estrogens to affect the physiologic functions of humans or other animals. As endocrine disruptors, they can be either synthetic or natural chemical compounds derived from diet, pesticides, cosmetics, plastics, plants, industrial byproducts, metals, and medications. By mimicking the chemical structure that is naturally occurring estrogen compounds, synthetic XEs, such as polychlorinated biphenyls (PCBs), bisphenol A (BPA), and diethylstilbestrol (DES), are considered the focus of a group of exogenous chemical. On the other hand, nature phytoestrogens in soybeans can also serve as XEs to exert estrogenic activities. In contrast, some XEs are not similar to estrogens in structure and can affect the physiologic functions in ways other than ER-ERE ligand routes. Studies have confirmed that even the weakly active compounds could interfere with the hormonal balance with persistency or high concentrations of XEs, thus possibly being associated with the occurrence of the reproductive tract or neuroendocrine disorders and congenital malformations. However, XEs are most likely to exert tissue-specific and non-genomic actions when estrogen concentrations are relatively low. Current research has reported that there is not only one factor affected by XEs, but opposite directions are also found on several occasions, or even different components stem from the identical endocrine pathway; thus, it is more challenging and unpredictable of the physical health. This review provides a summary of the identification, detection, metabolism, and action of XEs. However, many details of the underlying mechanisms remain unknown and warrant further investigation.

## 1. Introduction

As endocrine disruptors, xenoestrogens (XEs) are substances that imitate endogenous estrogens to affect the physiologic functions of humans or other animals. In the 1950s, some research demonstrated the hormone-like effects, mostly associated with environmental chemicals in wildlife. Either estrogenic xenobiotics or XEs are considered the major disruptors of endocrine, with a diverse chemical structure [1]. Exposure to not only nature, which phytoestrogens and mycoestrogens are the pertinent examples, but also industrial chemicals, estrogenic, and anti-estrogenic activity have been exhibited [2]. Through the imitations and obstacles in responses via the non-genomic and/or genomic signaling pathway, endogenous estrogens are largely disrupted by XEs [3]. Individuals are exposed to a complex mix of chemicals during their lifetime. XEs, which can be found in every aspect ranging from the environment, food, cosmetics, and other substances, have pros and cons to the human body [4,5]. By mimicking the chemical structure that is naturally occurring estrogen compounds, some XEs are considered the focus of a group of exogenous chemical, which can be derived from the sources as follows: diet, pesticides, cosmetics, some plastics, plants, fumes, industrial byproducts, metals and medications (such as oral contraceptives) [4,6,7,8]. The importance of new chemicals’ endocrine-disrupting potential cannot be ignored [1].

The estrogenic or antiestrogenic activity of chemicals is attributed to the interactions between estrogen receptor (ER) and other compounds. In fact, ER, as a ligand-inducible transcription factor, plays a vital role in development and neoplasia using regulating genes involved in cell proliferation and differentiation.

**Figure 1:**
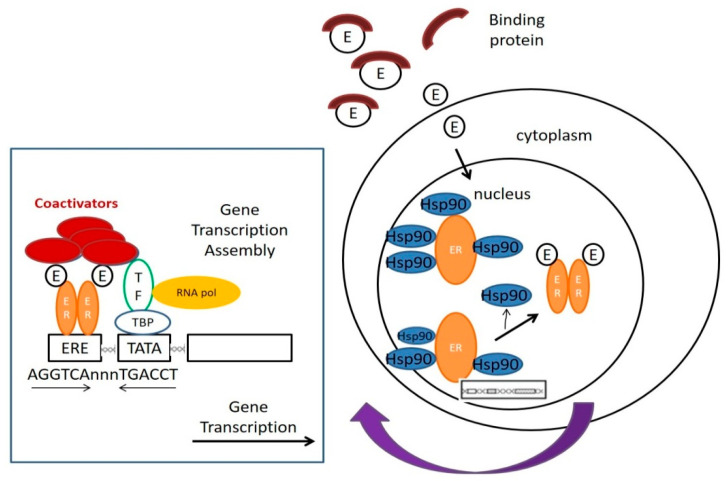
The molecular mechanisms for the actions of estrogen receptors [9,10,11].

Muellery has clearly defined XEs in the review article, which described the mechanisms of action and detection methods, stressing the points of molecular cell biological mechanism to estrogen receptor-mediated hormone actions. Figure 1 has displays how the estrogen or XEs work in cells through several consecutive steps [9,10,11].

(1.) Estradiol, a type of estrogen (E), is trapped over by carrier protein in the serum. After the estrogen is released from the blood by the carrier protein, it can pass through the cell membrane without disturbance and enter the cell;
(2.) Located within the nucleus, before activation, the ERs will be bound to various receptor-associated proteins, such as heat-shock proteins (Hsp90);
(3.) Estrogen will be bound to the ERs, replacing the receptor-associated proteins;
(4.) ER dimer is bound to its corresponding DNA-binding domains, and the sequence is named estrogen-responsive element (ERE);
(5.) Under the cooperation of assembled substances, including multiple transcription factors (TF), the RNA polymerase (RNA Pol) and other proteins, transcription can be started. The relevant mRNA sequence emerges as ER target genes transcription is in the process;
(6.) Co-activators, including CBP/p300 and SRC-1, are all the linking brackets to ER dimer and could play an effect on DNA transcription;
(7.) After DNA is transcribed into mRNA, RNA should be translated to produce protein to complete gene expression [10].

Like the ligand actions of endogenous hormones, the ER α and β shall be bound and activated before XEs bring their effects fully into play. There is not only one single factor affected by XEs, but opposite directions are also found on several occasions, or even different components stem from the identical endocrine pathway; thus, it is more challenging and unpredictable in terms of physical health. Interestingly, XEs are most likely to exert tissue-specific and non-genomic actions when the concentrations are relatively low. The debate on the risks that humans are exposed to remains a controversy, and a clear-cut relationship between XEs exposure and human health so far has only a little evidence. However, due to the complexity of their mechanisms and potential for adverse effects, how XEs affect normal estrogen signaling remains an open question and is worth investigating [12].

The estrogenic environmental compounds, including bisphenol A (BPA) and butyl benzyl phthalate (BBP), may trigger the adverse effects of endocrine-disrupting chemicals on organisms. As the natural estrogen, 17β-estradiol is the major factor in forming breast cancer and further to the progression. An in vitro- in vivo model has been developed to demonstrate the carcinogenicity of natural estrogen 17β-estradiol and xenoestrogenic substances in human breast epithelial cells MCF-10F. In this model, hypermethylation of NRG1, STXBP6, BMP6, SS3, SPRY1, and SNIP were found at different progression phases. Whether BPA and BBP are relevant to breast cancer initiation can be demonstrated using this unique model. The evidence mentioned earlier suggests that natural estrogen 17β-estradiol and xenoestrogenic substances, such as BPA, are considered to trigger a neoplastic transformation in human breast epithelial cells [13].

Ullah et al. have conducted an in vivo study and investigated whether chronic exposure to low doses of BPA and its analogs affects spermatogenesis with outcomes on oxidative stress and the male reproductive system of 22-day-old rats [14]. Oxidative stress in the testis was significantly elevated, while sperm motility was impaired. The daily sperm production and the number of sperm in the epididymis were reduced. This research confirmed that exposure to BPA and its analogs for a chronic duration could induce structural and functional changes in testicular tissue and endocrine alterations in the male rat reproductive system [14].

To investigate the effects of XEs on skeletal programming, Pelch et al. have compared the skeletal effects of low-dose BPA exposure to mice 9 days prenatal and 12 days postnatal in 2012 [15]. The skeletal health of these mice was assessed during adulthood when they had reached peak bone mass. The study revealed that exposure to 10 µg/kg/day BPA significantly increased the femoral length in the male mice but decreased the biomechanical strength in the female mice [15].

Research across the 1960s to 1970s demonstrated the estrogenicity of a couple of industrial compounds and pesticides, o,p-DDT, kepone, methoxychlor, phenolic derivatives, and polychlorinated biphenyls (PCBs). Several environmental chemicals have been categorized into the list of XEs, including the pesticide toxaphene, dieldrin, and endosulfan, and some different compounds used in the food industry, which are antioxidants, such as *t*-butylhydroxyanisole. The BBP and 4-OH-alkylphenols are components of plasticizers. The substance BPA was applied in dental restorations [1,5].

In Korach’s studies, it is indicated that even weakly active compounds could interfere with the hormonal balance with persistency or high concentrations of XEs. Congenital malformation of wildlife and humans born with birth defects are negatively affected by endocrine-disrupting chemicals XEs. The development of the urinary tract and nervous system is particularly sensitive to hormonal disruption over periods of in utero and early postnatal life [5,16]. On top of that, a birth defect can be considered permanent damage, whereas the structural changes of the body parts are less affected after reaching adulthood [12,17].

During the 1990s, male reproductive problems ranging from the decline in semen quality, testicular cancer, hypospadias, and cryptorchidism seem to be increasing health issues found in Belgium, Denmark, France, and Great Britain. In 1996, studies suggested that the supernormal levels of estrogens, specifically diethylstilbestrol (DES), were the leading cause of male fetus reproductive defects. Regarding common XEs stemming from environmental contaminants and chemicals, their adverse effects on male reproductive health are exceptionally crucial. The stages of fetus and childhood are more vulnerable than that of mature adulthood. An extensive study should explore the underlying problem further and strategically determine the intervention and potential treatment [18].

Estrogens can act on signaling pathways with pertinent examples in diseases, such as cancer, cardiovascular, metabolic, or immune system disorders. Many XEs gathered by natural and synthetic compounds can behave as estrogens and have a close interaction with ERs. These compounds are capable of bonding to the ER in the cell, leading to bioaccumulation as a whole [3,19,20,21]. In contrast, some XEs are not similar to estrogens in structure and can affect the physiologic functions in ways other than ER-ERE ligand routes. In fact, the high prevalence of XEs has brought tremendous interest in in-depth research concerning hormone-dependent cancers, including breast, ovarian, endometrial, prostate, thyroid, and cervical.

Between 1940 and 1971, nearly 2 to 4.8 million human offspring were exposed to DES, a strong synthetic estrogen. Research has concluded that prenatal women exposed to DES were subject to the growth of adenocarcinomas of the cervix and reproductive disorders [22,23,24]. Interestingly, DES has been applied in therapeutic hormone replacement for women, extensively using prostate and breast cancer treatment through having cancer-induced factors. On the other hand, DES also functions well to suppress the androgen action and induce apoptosis toward both androgen-dependent tumors and independent by interfering with the cell cycle. To sum up, XEs have their pros and cons, contributing differently to human beings and creatures [22,25,26]. Since XEs can act as endocrine disruptors to affect functions of humans and animals, how they can be detected and how they can act in the endocrine system are important to further studies. This overview provides a summary of the identification, detection, metabolism, and action of XEs.

## 2. Identification and Detection of Xenoestrogens

The literature revealed that XEs, as active endocrine substances, can interact with estrogen receptors, androgen receptors, or peroxisome proliferative receptors [12]. By inhibiting or activating these nuclear receptors, XEs can affect circulating hormone levels and disrupt normal endocrine function, thus possibly resulting in metabolic syndrome, reproductive dysfunction, and the occurrence of cancers [3,4,27,28,29]. The exposed concentration, dose, and time of XEs, as well as the age of contact persons, have different impacts on affected individuals [30].

In the past, there were various in vivo and in vitro assays developed to measure the estrogenic-like activity of XEs, as well as the concentration and potency of the estrogenic and antiestrogenic compounds. For a better understanding of morphological, histological, biochemical, and molecular actions, researchers have completed different studies to analyze the estrogenic properties of XEs. Although the in vivo real actions of XEs are unpredictable, many in vitro tests have been continually used to explore the estrogenic potency of XEs [9,31,32,33,34].

Studies regarding the identification and detection of XEs, and investigating the molecular mechanisms of interactions between XEs and ERs were solicited from the literature. They described in detail the characteristics of the estrogenic or antiestrogenic potency of different XEs. A summary of the results of these studies is shown in Table 1.

Mueller et al. [9] reported a review article in 2004, focusing on several in vitro tests for screening estrogenicity and antiestrogenicity of XEs. These assays were all powerful tools elucidating and describing the effects and mechanisms for XEs actions. While they had discussed in the essay, some mechanism-based assays are inappropriate to high-throughput screening for potent estrogenic and antiestrogenic XEs. Instead, other research [9,35], including high-throughput screening methods published recently, was presented. In vitro assays for identifying and detecting estrogenicity and antiestrogenicity toward different XEs were all summarized in Table 1.

### 2.1. E-SCREEN

Soto et al. [33] used the E-SCREEN assay to investigate the effects of XEs and 17β-estradiol on cells. Employing human MCF-7 (breast cancer cells) as a target in the assay, XEs were observed competing with E2 for binding to the ER and increased the levels of progesterone receptor (PR) and pS2 in MCF-7 cells. The E-SCREEN assay results have confirmed the estrogenic materials, including alkylphenols, phthalates, PCB congeners, and hydroxylated PCBs. The insecticides dieldrin, endosulfan, and toxaphene are also detected in the assay.

### 2.2. Ligand-Binding

In 1998, Randall Bolger et al. reported a novel technique for detecting XEs named the fluorescence polarization (FP) method. Under room air, the method could measure the capacity of XEs to displace a high-affinity fluorescent ligand from purified, recombinant human ERα. The ERs in mice have two subtypes, including ERα and ERβ, which are different in the structures of the C-terminal ligand-binding domain and N-terminal transactivation domain. In the mouse model, Kuiper et al. observed ERα- and ERβ-mediated messenger RNA expression via ligand binding and compared the specificity and affinity of ligand binding to ERα and ERβ via RT–PCR. They also found differences in the distribution and expression of ERα and ERβ in tissues and organs of mice. There were more distribution and expression of ERα in the uterus, testis, pituitary, ovary, kidney, epididymis, and adrenal, while there were more distribution and expression of ERβ in the prostate, lung, bladder, and brain [36,37].

### 2.3. ER-Binding to ERE

Nikov et al. observed differences in affinity of phytoestrogens when combined with ERα or ERβ, respectively. They also mentioned the combinations and interactions of ligand–ER complexes and estrogen response element (ERE) sequences. In their experiments, the FP methods were employed to measure binding affinities of various phytoestrogens, such as genistein, coumestrol, daidzein, glyceollin, and zearalenone to human cells. This article revealed a higher affinity of the phytoestrogens for ERβ than for ERα. The effect of these phytoestrogens on the ability of ERα and ERβ to associate with specific DNA sequences (EREs) was also investigated. In 2000, Boyer et al. performed a similar method on human ERα for probe of its molecular way on functional specificity. The implemented binding assays to study the interaction of the receptor with a palindromic estrogen response element derived from the vitellogenin ERE [38,39].

### 2.4. Mammalian Two-Hybrid Assays

DES is a well-known carcinogen [37]. As a mixture of indenestrol A (IA) S and R enantiomers [39], IA is a metabolic derivative of DES and has a high affinity to bind ER but a weaker biological activity [38]. Mueller et al. found that the estrogenic properties of the S and R enantiomers of IA, IA-S, and IA-R, respectively, had different affinities with ERα and ERβ in cells. Using human endometrial (Ishikawa) and breast MDA cell lines, which stably express either ERα or ERβ, IA-S was found more potent to activate cell transcription through ERα compared to IA-R. However, IA-R had more potency to stimulate ERβ rather than ERα in MDA cells, but this was not the case in endometrial cells. Although IA-R could effectively activate ERβ in vivo, it had a low affinity to bind both ERα and ERβ in vitro. These results showed that IA-R was cell-selective when bound to ERβ. In addition, there existed a single residue within the ligand-binding domains to determine the stereoselectivity of both ERα and ERβ [40,41,42].

### 2.5. Analysis of Gene Expression

Jorgensen et al. demonstrated that estrogenic activity could be evaluated by assaying induction or repression of endogenous estrogen-regulated “marker genes” in human breast cancer MCF-7 cells. The authors mentioned that a cell-based endogenous gene expression assay is very sensitive to what could be used to assay the estrogenicity of different putative estrogenic chemicals. They performed an assay in human MCF-7 that was estrogen-dependent and used a method of polymerase chain reaction (PCR) to observe the changes in gene expression. After differential display using reverse-transcribed (DDRT) PCR technology, the levels of expression could be quantified by phosphor-imaging [43,44].

### 2.6. Analysis of Enzyme Activity

Holinka et al. [46] described alkaline phosphatase as a convenient endpoint to examine mechanisms of hormonal actions. Alkaline phosphatase activity in human endometrial cancer cells of the estrogen-responsive Ishikawa line was markedly stimulated by estrogens, 5a-dihydrotestosterone (DHT), and dehydroepiandrosterone (DHEA). In the previous report, E2 and several other estrogens were noticed to greatly enhance the activity of alkaline phosphatase, an enzyme known to be regulated by ovarian hormones in the nonpregnant and pregnant rodent and monkey uterus. The results suggested that estrogen and androgen receptors mediate the stimulation of alkaline phosphatase by the C19 steroids [45,46].

### 2.7. Analysis of Steroido-Genesis

In H295R cells, various pesticides that had been suspected of interfering with the functions of steroid hormones were examined for their effects on the mRNA expression and catalytic activity of aromatase. Sanderson et al. focused mainly on interactions of XEs with sex hormone receptors, particularly the estrogen receptor. After a period of time, other mechanisms of interference with endocrine functions have gained attention, including the effects of pesticides on the enzymes that are involved in the synthesis and metabolism of steroid hormones. As a key role in producing many endogenous steroid hormones of high potency, the enzymes cytochrome P450 (CYP) that are responsible for the reactions in the biosynthetic pathways of steroids is a research focus [47]. H295R cell line was used to screen several pesticides known or suspected to interfere with steroid hormone function for potential effects on the catalytic activity and mRNA expression of aromatase [47,48].

### 2.8. The Fluorescence-Based Multi-Analyte Chip Platform

**Figure 2:**
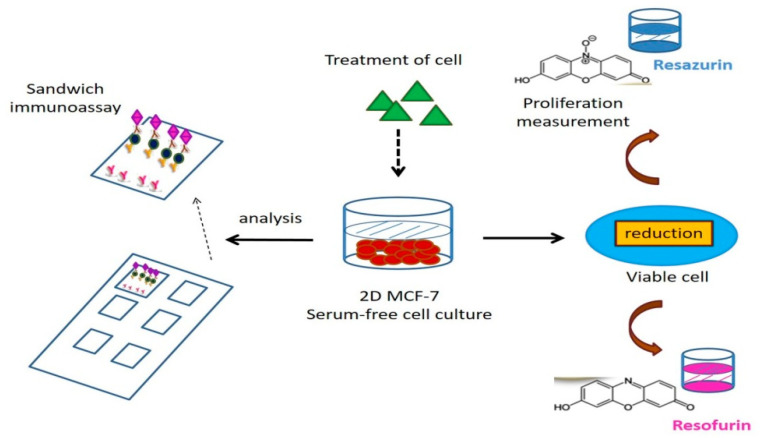
The fluorescence-based multianalyte chip platform. This equipment contains MCF-7 cells, which are exposed to estrogen receptor agonists and antagonists. Using a resazurin assay, the proliferative effect in hormone-sensitive cancer cell line MCF-7 can be measured. Using a multiplexed protein microarray with fluorescence detection, biomarkers can be quantified in the supernatant. By employing immobilized antibodies, the on-chip sandwich immunoassay captures the biomarker and a fluorescently labeled detection antibody for confirmation [52].

Using a parallel-proliferation measurement, the fluorescence-based multiplexed protein microarray (Figure 2) presents the bidirectional estrogenic and anti-estrogenic actions of XEs in human MCF-7 cells [52,53,54,55]. As a new in vitro tool for screening environmental samples, the fluorescence-based chip platform can analyze multiple targets to detect the estrogenic and anti-estrogenic actions of XEs by observing the expression of proteins. It can quantify 10 proteins secreted by MCF-7 cells, which represent different physiological and pathological endpoints of endocrine actions. Its potential is demonstrated by distinct protein secretion patterns of the cancer cell line after exposure to known ER agonists ß-estradiol, BPA, genistein, and nonylphenol as well as antagonists fulvestrant and tamoxifen. In parallel, the proliferating effect of endocrine-disrupting substances in MCF-7 cells can be assessed in a proliferation assay based on resazurin. Unlike the detection tools developed earlier, the chip has two advantages, including high-output screening for the endocrine effects of environmental disruptors on human cells and providing messages of the complicated cellular effects on a molecular level. Compared with single marker detection, this multiplexed protein microarray in the chip is more convenient and arrives at better accuracy [52].

### 2.9. Transcriptional Activation Assays

A plasmid vector contains firefly luciferase genes that are controlled by DNA enhancers, which can react with androgens, estrogens or retinoic acids. The transfection of a plasmid vector into corresponding receptor-containing cells reflects its ability to respond to respective hormones under luciferase induction. An estrogen-responsive luciferase reporter plasmid has steadily transfected the recombinant ovarian carcinoma (BG-1) lines in humans. The subsequent recombination of cell lines (BG1Luc4E(2)) can respond to 17β-estradiol of a low concentration (≤1 pM). As a screening system for environmental hormones, the detectivity of BG1Luc4E(2)-cell bioassay was identified by the individual response to common XEs, and also by two novel estrogenic chemicals of PCBs (2,3′,4,4,′-tetrachlorobiphenyl and 2,2′,3,5′,6-pentachlorobiphenyl). These cell bioassay systems have applications for rapid screening, identification, and characterization of endocrine-disrupting chemicals. Transcriptional activation assays allow rapid identification of compounds with the potential to affect the ER signaling pathway directly or indirectly [56].

### 2.10. Triple Functional Small-Molecule–Protein Conjugate-Mediated Optical Biosensor

It is a challenge to create biosensors for a comprehensive mapping of potential estrogenic chemicals. Using triple functional small-molecule–protein conjugates as probes, fluorescent ER-based-wave biosensors were reported in 2019 to detect estrogenic activities in water samples [57]. The probe containing a Cy5.5-labeled streptavidin (STV) part and a 17β-estradiol part can act as signal conversion and recognition. When XEs are competing with the E2 part of the probe in binding to ERs, the unbound conjugates will be released. The STV parts then bind with desthiobiotin (DTB) modified with the optical fiber through an STV-DTB affinity interaction. The detection with signal probes is completed by fluorescence emission induced by a descending field, which correlates with estrogenic activities in the samples.

A facile method for quantifying estrogenic activities is developed by using a triple functional small-molecule–protein conjugate as a sensing element. Following optimization of detection, exposure to environmental estrogens can result in the release of the fluorescein-labeled conjugate in the supernatant. It will increase the amount of fluorescein that is attached to the fiber surface, which can be observed by increases in the fluorescent signals. By means of the approach, samples’ estrogenic activities can be measured at a limit of detection (LOD) of 1.05 μg/L, using E2 as a reference. The biosensors provide a reliable application for the detection of estrogenic activities in real samples of wastewater [57].

### 2.11. Estrogen Receptor Recombinant Yeast Screening (YES) Assay

Described by Professor Sumpter, this estrogen receptor recombinant yeast screening (YES) assay can be used to assess the estrogenic activity of chemicals or their metabolites, such as the surfactants and their major degradation products. In principle, the DNA sequence of the human estrogen receptor (hER) is integrated into the yeast *(Saccharomyces cerevisiae*) genome, which also contains expression plasmids carrying estrogen-responsive sequences (ERE) that control the expression of the reporter gene *lac-Z* (encoding the enzyme β-galactosidase). Thus, in the presence of estrogens, β-galactosidase is subsequently synthesized via the formation of ER-ligand and secreted into the medium, where it causes a colorful change from yellow to red [58]. This recombinant yeast is used to determine whether chemicals or their metabolites, such as the surfactants and their principal degradation products, possess estrogenic activity. The results are compared to the effects of the main natural estrogen 17β-estradiol [58]. Using the YES assay, the study revealed none of the parent surfactants tested possessed estrogenic activity. However, one class of surfactants, the alkylphenol polyethoxylates, degrade to persistent metabolites that were weakly estrogenic. Another group of degradation products, the sulfophenyl carboxylates, which were derived from the biodegradation of linear alkylbenzene sulfonates, did not appear to possess estrogenic activity [58]. Hence, the estrogen receptor recombinant YES assay can evaluate the estrogenic stimulation provided by estrogens, XEs and other endocrinal disruptors.

## 3. Metabolism and Action of Xenoestrogens

### 3.1. The Metabolism of Xenoestrogens

The estrogenic or antiestrogenic activity of chemicals is attributed to the interactions between ER and other compounds. In fact, ER, as a ligand-inducible transcription factor, plays a vital role in development and neoplasia, regulating genes involved in cell proliferation and differentiation [9].

As a role of the major transcription factor in cell proliferation and differentiation, ER is sensitive to any disruption of the signaling pathways, leading to infertility, developmental abnormalities, or endocrine cancer discovered in both human beings and wildlife. The damage to health may be originated from exposure to the estrogenic or antiestrogenic activities of chemicals [59]. Endocrine-active compounds may also interfere with other signaling systems, most significantly the androgen and thyroid hormone system, steroidogenesis [60], and part of the aryl hydrocarbon (Ah) receptor [61].

The ER, as the nuclear receptor superfamily, is a factor of ligand-inducible transcription. Hence, far, two subtypes of the ER are ER α [62,63,64,65] and ER β [64], respectively, and both receptors have unique tissue distribution, playing a significant role in physiology [66,67]. In 1999, Korach suggested that most aberrant phenotypes are proved to be linked to ER α from the ER knockout mice study. (e.g., hypotrophy of the uterus, infertility, and rudimentary mammary gland development) [67].

While the reproductive system is significantly affected by the ER α, another impact upon non-classical estrogen target tissues, such as brain, skeletal, immune, cardiovascular system, adipose tissue, and the male reproductive tract, is also crucial [66,68]. As opposed to ER α, with diverse influences on multiple systems, ER β impacts are more specific to the female ovarian function [69,70]. Therefore, more attention has been paid to the XEs in which the dominance of ER α is much more than ER β; however, the interesting findings indicate that than ER α, ER β has correctly shown higher affinity [10,71].

Researchers have made attempts to reach the conclusions of the distribution and locations of the ER through the study of mice or even human body tissues. In 2001, Mueller and Korach et al. demonstrated the RNA and protein expression of ER in the human body [10,70]. There were published data regarding the distribution of relative qualitative tissue expression of ER α and ER β on cells and tissues of humans and mice [37,72,73,74,75].

Estrogen contributes largely to cells in various aspects, including development, proliferation, migration, and survival [67,76]. The action can be divided into two, namely genomic and non-genomic pathways [76,77]. Receptors, such as ER α, ER β, or even GPER, function well through these pathways [11,78,79]. The binding of estrogens to ERs in the cytoplasm contributes to the genomic pathway. The estrogen/receptor complex then swift to the nucleus; thus, changes are made to gene expression [80].

Additionally, non-genomic pathways, as influential as they could be, are via the binding to receptors (GPER) in cell membranes to activate secondary cellular messengers without incorporating gene expression in the nucleus. While both pathways are essential to further actions, non-genomic pathways, with their unique feature, have made the processing time faster from seconds to minutes; in contrast, genomic pathways are considered less efficient as it could take from an hour up to a few days for the transmission of messages in the organism [76,77].

The ER is located within the nucleus and dimerizes on ligand binding, and then the ER dimer is bound to the estrogen response element (ERE), which is in the sequence of the promoter of the estrogen target gene, being called the ER transcription complex. Dimers, including ER, ER α or ER β homodimer and ER α /ER β heterodimers are capable of binding to the corresponding ERE, which in turn prompts gene expression. Unlike ER β homodimers, ER α/ER β heterodimers seem to be more intensive in all ER activities. Additionally, ER α is discouraged by the existence of ER β activity [81,82]. The factors that accelerate gene transactivation are known as coactivators [79,83]; however, DNA is suppressed by the corepressors [84,85]. Both coactivators and corepressors are defined as the provider of the ER action.

The most common classical mechanism is displayed in Figure 1. Nevertheless, channels that will affect ER would not only be considered a single classical pathway but also the multiple interveners [11]. Moreover, then, the Kinase cascades or ER phosphorylation may be signaling information toward the ligand-independent activation. To be more specific, the estrogen ligand is connected to the ERE in which the signal of E2-target genes can be clearly expressed, such as the ER-DNA interaction classical pathway [12]. However, not all of the ERE would be found in the E2-sensitive gene promoter sequence, implying that the ER modes of action are diverse rather than a simple pathway.

Almeida et al. reported that ERα in osteoblast progenitors expressing Osterix1 (Osx1) potentiates Wnt/β-catenin signaling, thereby increasing proliferation and differentiation of periosteal cells [86]. Further, this signaling pathway was required for optimal cortical bone accrual at the periosteum in mice. Notably, this function did not require estrogens. The osteoblast progenitor ERα mediated a protective effect of estrogens against endocortical but not cancellous bone resorption. ERα in mature osteoblasts or osteocytes did not influence cancellous or cortical bone mass. Hence, the ERα in both osteoblast progenitors and osteoclasts functions to optimize bone mass, but at distinct bone compartments and in response to different cues [86].

In another study, it was hypothesized that ERα in osteocytes was important for trabecular bone in male mice and for cortical bone in both males and females [87]. Dmp1-Cre mice were crossed with ERα(flox/flox) mice to generate mice lacking ERα protein expression specifically in osteocytes (Dmp1-ERα (−/−)). Male Dmp1-ERα (−/−) mice displayed a substantial reduction in trabecular bone volume (−20%, *p* < 0.01) compared with controls. ERα in osteocytes regulates trabecular bone formation and thereby trabecular bone volume in male mice, but it is dispensable for the trabecular bone in female mice and the cortical bone in both genders. The authors proposed that the physiological trabecular bone-sparing effect of estrogen is mediated via ERα in osteocytes in males but via ERα in osteoclasts in females [87].

Kapara et al. have adopted a nondestructive approach for detecting and localizing ERα expression at the single-cell level using surface-enhanced Raman spectroscopy (SERS) combined with functionalized gold nanoparticles (AuNPs) [88]. The author developed an approach based on the percentage area of SERS response to qualitatively measure expression level in ERα-positive (ERα+) breast cancer cells. Specifically, the calculation of relative SERS response demonstrated that MCF-7 cells (ERα+) exhibited higher nanotag accumulation resulting in a 4.2-times increase in SERS signal area compared to SKBR-3 cells (ERα-) [88]. The result of this article confirmed the strong targeting effect of ERα-AuNPs towards the ERα receptor. It opened up the possibilities of using SERS as a tool for the estimation of ERα expression levels without the requirement of destructive and time-consuming techniques. Therefore, the potential of using SERS as a rapid and sensitive method to understand the activity of SERDs in breast cancer is demonstrated [88].

ER β has been suggested to possess antiproliferative and antitumor effects in breast and prostate cancer cells in some previous articles, but other studies have indicated its tumor-promoting effects [89]. The author studied the effects of ERB-041-treated colon cancer cells in a zebrafish xenograft model and found significantly less distant metastasis of ERB-041-treated cells compared to vehicle-treated cells. These results further support ERβ’s antitumor role in colorectal cancer and the possible use of its agonist in colorectal cancer patients [89].

Estrogen-related receptor β (ERRβ) is a nuclear receptor critical for many biological processes. Despite the biological and pharmaceutical importance of ERRβ, deciphering the structure of ERRβ has been hampered by the difficulties in obtaining a pure and stable protein for structural studies [90]. In fact, the ERRβ ligand-binding domain remains the last unsolved ERR structure and also one of only a few unknown nuclear receptor structures. The authors confirmed a critical single-residue mutation resulted in robust solubility and stability of an active ERRβ ligand-binding domain, thereby providing a protein tool enabling the first probe into the biochemical and structural studies of this important receptor [90].

The promoter context and estrogenic ligands are the joint dependents upon the expression of ER-target genes and ER-mediated cellular functions. In the previous discussion regarding the classic pathway, estrogenic ligands are highlighted as the trigger of ER conformational changes, and with further interactions with other coregulators and subsequent transcriptional activity moving forward [91,92].

It is indicated that the XEs work in different functions, even if they are identical. The tissue specificity marks its uniqueness, and XEs could also turn to other tissues with complexity. As ERα and ERβ have different functions and effects, the physiological functions and the organs and tissues are also distributed differently in the body parts; thus, identical XEs could lead to different consequences and effects on the body. Generally, XEs are either partial agonists or partial antagonists, which indicate that they are possibly less effective than estrogen in the human body [9].

As mentioned above, the complexity of mechanisms that affect XEs in the human body includes the genomic pathway, non-genomic pathway, and various transcription factors. Hereafter, coactivators, corepressors, or even the interactions between signaling cascades and other receptors will lead to estrogenic effects. Therefore, the mechanisms mentioned above should be considered in the experiment and evaluation. The diversification of ER action is the key to be understood [93].

In recent decades, people became aware of the exogenous compounds that work on the signaling pathways of endogenous hormones in the human body or organisms, including synthesis, storage, metabolism, transport, and elimination in organisms [94,95].

As endocrine-disrupting chemicals (EDCs), these XEs may lead to biological and pathological changes or even accumulated damage to future generations (e.g., bioaccumulation). There are various sources and types of XEs in the environment, and many of these XEs have the similarity of structures with estrogens [19,96]. The chemical structural similarity between these XEs and naturally occurring estrogen compounds can cause the human body and organism simulations (e.g., 17β-estradiol). EDCs behave like natural (estrone (E1), E2, estriol (E3)) and synthetic estrogens DES, [4,7]. Among all estrogens, including E1, E2 and E3, E2 is the most potent and serves as the major estrogen that exerts endocrinal functions in human bodies.

The mechanisms of action of XEs could be divided into critical factors as follows: Endogenously occurring estrogens simulation, an antagonist of endogenous estrogens, and the intervention of metabolism and biosynthesis production of estrogen [8]. Additionally, EDCs have been mentioned to act through complex tissue-selective modulation on ERs and other signaling pathways in vivo [19].

As mentioned above, ER α and ER β act as an intermediary in various tissues and those with distinct biological effects, including mammary glands, bone, brain, and vascular system in both genders. Due to the partial different tissue distribution and distinct physiological functions, XEs could show agonist or antagonist activity at the time of development [94].

The relationship between ERs and coactivators/corepressors is critical to the regulation of DNA and RNA, which will also impact the expression of ER-target genes. In fact, the characteristic of tissues specific to different organs includes expression of specific cofactors, the ER α/ER β ratio, and the level of expression of specific intracellular kinases (including cytoplasmic tyrosine kinases). For example, the affinity of the phytoestrogen genistein to ER β is much greater than that of ER α. There is even more evidence to show that genistein impacts the proliferation or antiproliferation in cancer cells [97].

### 3.2. The Actions of Xenoestrogens

#### 3.2.1. Selective Estrogen Receptor Modulators (SERMs) and Aromatase Interferer

Due to the characteristics mentioned above, tamoxifen and raloxifene have been applied to the current treatments, and the process is so-called selective ER modulators (SERMs). These kinds of XEs sometimes could have impacts on the ER nongenomic pathways that increase endocrine disruption. There were examples showing XEs in different structures, with high concentrations, BPA, and DES, all capable of activating ERs, thus increasing the risk of developing breast cancer [98].

SERMs were found to demonstrate selectivity toward ERs in the bone, thereby reducing side effects. However, they lack the efficacy of traditional estrogen. SERMs are generally influenced by their binding affinity for ER α and ER β and the effect of the bound ligand on the ER structure. However, the precise mode of action of each SERM remains unknown. One endogenous compound—27-hydroxycholesterol (27HC)—has been found to bind to and modulate the activity of ERs in vivo and to behave like a SERM. In mice, 27HC behaves as an ER antagonist and reduces the protective effects of estradiol. However, in cellular models of ER-positive breast cancer, 27HC acts as a partial ER agonist. In ovariectomized mice with elevated 27HC levels, a dramatic loss of bone was observed. Further research on 27HC could lead to developing new drugs.

Aromatase inherently existed in the human body to convert androgens to estrogens. However, a type of XE, such as tributyltins, has an aromatase suppressing effect, leading to an imbalance between androgens and estrogen. Tributyltins is a kind of coating, which often exists in the hull of fishing boats or plastic products, and the potential health effects on organisms are as follows: teratogen; teratogenicity; diabetes mellitus; hyperlipidemia; metabolic syndrome; increase in fat depot size; obesity; hepatic steatosis; hypertrichosis; osteoporosis; decreased sperm production; breast cancer; endometrial cancer [99,100]; polycystic ovary syndrome (PCOS) [101,102,103,104].

#### 3.2.2. Polycyclic Aromatic Hydrocarbons (PAHs)

Some XEs are combined with sex hormone-binding globulin, which decreases E2 plasma transport in the cell. An example is an interaction between polycyclic aromatic hydrocarbons (PAHs) and ERE-dependent E2-target gene transcription. PAHs are a collective term for more than a hundred different chemical substances, which are formed when coal, fuel oil, gas, trash, or other organic substances are incompletely burnt, and some PAHs are artificially manufactured. PAHs are found in coal tar, crude oil, and a few are applied in medicines or manufacturing dyes, plastics, and pesticides [105,106,107]. On the other hand, some metabolites of PAHs could be combined with ERs to recruit other coregulators, and the enhancing performance of E2 target genes could be achieved. Dioxin is one of the metabolites of PAHs. In the case of dioxin, it could be bound with aryl hydrocarbon receptor (AhR) that leads to heterodimerization along with aryl hydrocarbon nuclear translocator (Arnt). If so, the interaction between ER and ERE could facilitate the expression of the E2 target gene through such a complex [94].

#### 3.2.3. DDT [2,2-Bis(p-chlorophenyl)-1,1,1-trichloroethan] and Its Metabolite DDE

Another example of XEs is DDE, a metabolite of DDT and a component of pesticides. DDE is seen as more effective due to its affinity and lipophilicity, and it is unlikely to be catabolized by organisms. Both DDT and DDE are XEs with estrogenicity, which could influence the reproduction function of organisms in the environment. For instance, in the alligators of Lake Apopka, biologists have discovered that male crocodiles have a micropenis and various abnormalities of the testes due to their exposure to DDT and DDE substances [108].

Fujisaki et al. have reviewed past data records from the early 1980s that the American alligator (*Alligator mississippiensis*) population decreased in Lake Apopka after DDT and DDE exposure. Thus, the US government has conducted the extensive restoration of the swamp of Lake Apopka and proposed environmental-related restrictions on the lake in response to such a decrease. According to monitoring by the Florida Fish and Wildlife Conservation Commission, the adult alligator population gradually began to increase in the early 1990s after such efforts were made [109].

The consequences of DDT and DDE have also been found in human bodies; this was in Wolff et al.′s findings in the organochlorine article in 1993, including DDT, PCBs and other substances in relation to the risk of breast cancer. The correlation between breast cancer and DDT or DDE was mentioned [12,110].

Due to the inefficiency of metabolism and their solubility in lipids, these agents have been found in human tissue that causes lifelong sequestration in adipose tissue. Wolff et al. have concluded that DDE in serum is the most significant trigger of breast cancer development instead of associating with PCBs. This research suggests that environmental chemical contamination with organochlorine residues may be a critical factor in breast cancer [110].

In addition to the impacts of breast cancer, there is also evidence that shows methoxychlor (pesticide) and DDT have the potential to cause uterine proliferation and weakening of normal follicle development in the female reproductive system [19].

#### 3.2.4. Polychlorinated Biphenyls (PCBs)

Although Wolff et al. in 1993 reported that breast cancer was less associated with PCBs level in serum, other studies have suggested that PCBs work the same way in humans as scientists have long observed the effects of PCBs on other organisms. Bergeron noted the relationship between turtle sex determination and environmental contamination and the EDCs, such as PCB isomers, can alter sex ratios in turtles [12,111].

In the early 1970s, the findings in the experiments regarding PCBs in mice showed that exposure at birth reduced the reproductive ability of male rats [112] because of the alternation of steroid hormone-metabolizing enzymes [113]. Although Wolff et al. concluded that breast cancer was not associated with PCBs in serum in the early days, there have been many reports about PCBs and breast cancer progression in the future. Exposure to PCB174 has been confirmed to be associated with an increase in breast cancer mortality, and it is still positively related to breast cancer-specific mortality after 5 or even 15 years of follow-up after being diagnosed. Moreover, there are various reports, which made similar conclusions [93,114].

#### 3.2.5. The Relationship between EDCs and Diseases

It has been realized that estrogenic or antiestrogenic effects of different EDCs involve environmental pollution and affect human hormonal discrepancies. According to the historical data, breast cancer incidence and prevalence have been increasing since the 1940s [12,115,116,117]. The risk of breast cancer accelerates with increased cumulative estrogen exposure or the rise of XEs in the environment. Many researchers are dedicated to identifying related risk factors, DDT and its metabolite (metabolite) DDE [110]. As PAHs, dioxin, PCBs and DES were mentioned earlier, and all have been reviewed in the literature for the associated carcinogenic effects (carcinogenic effects, carcinogenic elements) or toxicity to the mammary gland (toxicant). 2,3,7,8-chlorodibenzo-p-dioxin has been proved to be toxic to the mammary glands of mice. Dioxin delays the proliferation and differentiation of the mammary glands of breast development. This finding has an identical conclusion obtained in the human body [118]. Another study reported that 200 young Belgian girls had delayed pubertal development related to their blood doubling of serum dioxin levels [119]. Similarly, PAHs have also affected a significant increase in postmenopausal women’s breast cancer development. Additionally, DES exposure during human pregnancy can trigger oncogenesis in the vagina and breast [120,121,122]. Moreover, Hoover proposed in 2011 that mothers who have been exposed to diethylstilbestrol will influence their next generation in utero, with the incidence and progress of future breast cancer development. In fact, the growth may become more significant due to the age of these DES daughters [123].

In 2016, Ellinon Axiomaticon reviewed studies that investigated the relationship between endometriosis and endocrine disruptors. Hormonal influences may be the key factor that affects the extrauterine growth and proliferation of endometrial cells [12]. Other reports demonstrated the association of membranous ectopic diseases of rodents or primates with the common environmental pollutants, including TCDD (2,3,7,8-tetrachlorodibenzo-p-dioxin), dioxin, BPA, and DES [124,125,126,127,128]. For the pathophysiology in relation to endometriosis that TCDD may cause, changes in the relative levels of ERβ and ERα in endometrial tissue determine the performance of the estradiol-regulated progesterone receptor (PR). The reduction of the ERα-to-ERβ ratio may result in the expression of PR being suppressed [129,130]. If the mother is exposed to specific-XEs, such as DES or BPA during pregnancy, increased endometriosis will be passed into the next generation, either in the human body or mice [125,127,131].

Similarly, endometrial cancer has the same trigger for developing ovarian cancer, which is considered estrogen and XEs [132,133]. For instance, methoxychlor [MXC; 1,1,1-trichlor-2,2-bis(4-methoxyphenyl) ethane] is an organochlorine pesticide used in agriculture since DDT was banned. The metabolite of methoxychlor is 2,2-bis(p-hydroxyphenyl)-1,1,1-trichloroethane (HPTE), activating ER in ovarian cancer cells and mitogenic activities in ovarian tissues. Moreover, triclosan is often used as a common ingredient in soaps, deodorants, toothpaste, and other hygiene products. Methoxychlor and triclosan are substances that contain organochlorine, regulating cell cycles and apoptosis-related genes by combining with ERs, thereby stimulating the growth of ovarian cancer cells and the consequences of cancer reaction [134]. In addition, genistein, which is a type of phytoestrogen, is similar in structure to estrogen, thus mimicking E2 and stimulating cell proliferation activity [135].

#### 3.2.6. Bisphenol A (BPA)

Industrial compounds, such as BPA and 4-nonylphenol (NPH), have residues in food (e.g., in canned vegetables) or dental materials. BPA is a chemical compound used in food or plastic containers. The coating on metal cans could protect food and beverages. Potentially, the residual compounds may all be detected [4,136,137,138].

If the mother mouse is exposed to the environment with BPA before delivery, the number of precancerous lesions will increase after the next generations turn into adulthood, and such correlation can be considered dose-related. However, high dosage BPA and 4-NPH can induce the occurrence of breast cancer cells in situ [137,139,140]. The possibility of XEs increase the risk of carcinogenesis in different vertebrate reproductive systems has been discussed. Moreover, current scientific and medical research has found that the impact of these environmental pollutants has been subjected to cancer-related and metabolic diseases, such as obesity, diabetes mellitus, hypertension, and cardiovascular disease [141,142].

Low levels of estrogen are related to developing glucose intolerance and insulin resistance [143]. As is, BPA can increase or decrease insulin production in the body by mimicking the effect of the body’s endogenous estrogen, which mechanism is similar to insulin regulation by 17β-estradiol. Recently, different research has proved the effects of BPA on insulin resistance, both for children and adults. BPA, as an environmental hormone, plays an important role in the pathophysiology of diabetes mellitus [144,145,146]. In addition, BPA can also reduce glycogen synthesis, thereby reducing glucose oxidation and reducing the use of glucose by muscle cells, and the sensitivity of muscle cells to insulin will be decreased [147].

BPA Exposure can also contribute to weight gain in mice, especially in female mice; the para-physiological mechanism may result from interference with the neurotransmitter signaling pathway, which causes the change in energy metabolism [142,148]. A study researching children’s obesity has confirmed that compared with normal weight, overweight and obesity are significantly associated with the urinary BPA levels of the children. Moreover, BPA may trigger insulin resistance in children and increase the risk of diabetes mellitus, especially for obese children [149]. In 2017, Lidia Caporossi conducted a literature review to analyze the effects of BPA on different metabolic diseases. Most of the reports are cross-sectional studies [141]. Fénichel pointed out that the prevalence of type-2 diabetes in the world has increased dramatically in the last few decades. There is also increasing evidence that shows these EDCs may also play a key role in the occurrence of metabolic diseases. In the observations of rodents, it was found that BPA stimulated the production and secretion of pancreatic β cells, interfering with insulin signals, which cause insulin resistance and β cell destruction/damages [150].

In addition to the risks regarding BPA that may result in various types of reproductive organ cancers and metabolic diseases, recent studies have also suggested that BPA can affect healthy bones. Except for the characteristics of estrogenicity and antiandrogenicity, it has been hypothesized that BPA can bind to the ER and exert its antiandrogenic, inflammatory, and oxidative properties [151]. Because bones will transform in response to the stimulation of hormones, inflammatory and oxidative status, BPA exposure can impair bone health. In 2018, Chin et al. reviewed the evidence of the effects of BPA and its derivatives on skeletal health in humans and animals and reported that BPA would decrease the proliferation of osteoblast and osteoclast precursor cells and induce apoptosis [151]. In various (in vivo and in vitro) animal models, BPA and its derivatives have different positive and negative actions. While BPA increased femoral bone mineral content in male rats, it decreased femoral mechanical strength in female rats. In estrogen-deficiency models, BPA improved bone mineral density and microstructures in aromatase-knockout mice; however, it lowered the trabecular density in ovariectomized rats. The major limitations of current evidence are the small sample size and the cross-sectional rather than longitudinal study design. In conclusion, BPA can affect the skeletal health of vertebrate animals, and its impacts depend on the types and sex of animals. However, the effects of BPA on bone mineral density and bone health of humans warrant further investigation [151].

Another study conducted by Kim et al. had attempted to analyze the relationships between serum BPA concentration, bone mineral density (BMD) and biochemical bone markers in postmenopausal women with osteoporosis [152]. The relationship between BPA and clinical variables was analyzed by the Pearson’s correlation test and the Kruskal–Wallis test. Serum BPA concentration was measured by enzyme-linked immunosorbent assay (ELISA). The mean BPA concentration of 51 postmenopausal women was 1.44 ± 0.52 ng/mL. The results showed no statistically significant correlation between BPA concentration and clinical variables. The author concluded that there was no statistically significant correlation between serum BPA and clinical variables related to bone metabolism [152].

Upson et al. have conducted a population-based case-control study to investigate the association of BPA exposure with the risk of endometriosis [153]. The authors measured and analyzed urinary BPA concentrations for more than 400 cases. For cases and controls, the median creatinine-uncorrected total BPA concentrations (µg/L) were 1.02 (IQR: 0.43–2.12) and 0.86 (IQR: 0.36–2.01), and the median creatinine-corrected total BPA concentrations (µg/g) were 1.32 (IQR: 0.79–2.21) and 1.24 (IQR: 0.65–2.54), respectively. The result showed statistically significant positive associations when evaluating total urinary BPA concentrations was only in relation to non-ovarian pelvic endometriosis, but not in relation to ovarian endometriosis [153].

Except for the association with female reproductive diseases, including breast cancer, ovarian cancer, vaginal cancer and endometriosis, literature has confirmed the effects of XEs on the male genital system. Increasing evidence has shown the connection between exposure and EDCs and impairment of male reproductive function. The impact can originate from the interference of hormone, cell signaling pathway and metabolism, and will be augmented, especially if the exposure to XEs occurs during early growth development [154]. Recent research has highlighted the possible relationship between unexplained male infertility and exposure to low-dose EDCs in the fetal testis and adult endocrine system [155]. Exposure to EDC may result in the impairment of testis functions in different spermatogenesis stages, depending on the time point of exposure. BPA induced meiotic abnormalities in the reproductive system of adult male mice [156] and significantly reduced the number of sperms in juvenile male mice as a result of interruption of meiotic progression [157,158]. Dibutyl phthalate (DBP) and methoxychlor (MXC) can significantly lower the weight of the testes as a consequence of a reduction in the number of spermatogenic elements and spermatozoa [159,160]. The use of DBP can block spermatogenesis, thus prohibiting the production of sperms and even leading to necrosis of the seminiferous tubules [159,160].

#### 3.2.7. Heavy Metals: Cadmium (Cd) and Arsenic (As)

Heavy metals can act as XEs and influence female reproduction systems. One example is the chemical Cd, which can be found in cigarettes, paints, plastics, batteries, and foods. As toxins to humans, inorganic Cd and As are cytotoxic at a high concentration level [4]; however, they can simulate estrogen and exert XEs-like actions at a low concentration level. Although the literature has confirmed the estrogenicity of inorganic Cd in tumor cell lines, the underlying mechanisms remain unclear and need more research. Both inorganic Cd and As stimulate cell proliferation in the pituitary gland and uterus by increasing the expression of proliferation markers, thus affecting hormone-dependent tumor progression. The anterior pituitary gland is responsible for hormone synthesis and secretion (such as prolactin and luteinizing hormone). Both inorganic Cd and As can increase prolactin synthesis. In 2016, Ronchetti et al. found that low doses of Cd can exert strong xenoestrogenic effects on the anterior pituitary gland [161].

#### 3.2.8. Phytoestrogens

Phytoestrogens are another type of XEs, including isoflavonoids, lignans, coumestans, and pisatin [24]. Some plants, such as soybeans, contain phytoestrogens with active ingredients of genistein and daidzein [4,162]. Phytoestrogens can combine and activate estrogen receptors in the brain to influence functions of the brain, thus possibly resulting in neurobehavioral disruptions. On the other hand, other studies indicated that pregnant women might benefit from the intake of soybean foods that contained phytoestrogens [163].

In vitro or in vivo studies to analyze the final effects of phytoestrogens may be quite different. For instance, at low doses (from 10 nM to 1 µM), genistein showed mitogenic effects on breast cancer cell growth, whereas, at higher concentrations (>10 µM), it showed antiproliferative effects [164]. Some of these effects are explained by their interactions with ER subtypes. As mentioned above, the ratios and the expressions of ERα and ERβ are different in various tissues depending on the period of life. Moreover, the abilities of ER subtypes to recruit cofactors, regulate gene expression and stimulate or inhibit cell growth are slightly different. Therefore, in vivo, phytoestrogens may have a complex role, acting as weak estrogens and antiestrogens depending on the tissue. Furthermore, it is believed that the signaling pathways induced by phytoestrogens are not completely identical to those induced by estrogens [164].

Regarding the preventive effects on diseases or cancers, a higher intake of phytoestrogens, such as isoflavones, is associated with a moderately lower risk of developing coronary heart disease. It may also reduce the risks of breast and colorectal cancer as well as the incidence of breast cancer recurrence. Consumption of phytoestrogens or soy foods is associated with reduced risks of endometrial and bladder cancer [165,166]. Regarding the therapeutic effects on menopausal syndrome or other diseases, phytoestrogens have been found to alleviate vasomotor syndromes even after considering placebo effects, reduce bone loss in the spine and ameliorate hypertension and in vitro glycemic control. They may also alleviate depressive symptoms during pregnancy. On the other hand, phytoestrogens have not shown definitive effects regarding improving cognition and urogenital symptoms [165,166]. Because of lacking standardization in the study designs, such as the ingredients and doses of phytoestrogens and the durations and outcomes of trials, it currently remains difficult to draw overall conclusions for all aspects of phytoestrogens. These limitations warrant further investigations of the use of phytoestrogens for women’s health.

#### 3.2.9. Diethylstilbestrol (DES) and 17-α-ethinylestradiol

DES is one type of synthetic estrogen with strong potency. During 1940–1971, several million people were exposed to DES [21,22,167]. DES can inhibit the actions of adrenal androgens and possibly interfere with cell cycles to induce apoptosis of prostate cancer cell lines [22,25,26]. A study from Egypt pointed out a higher incidence of uterine, cervical, and ovarian cancer in urban areas than in rural areas. The incidence of uterine cancer is 6-fold in urban areas than in rural areas. Correspondingly, there was a higher exposure to XEs (e.g., DES) for females living in the urban areas of Egypt than those living in rural areas [136]. Such a difference in exposure to XEs between urban and rural areas could be noted in other countries [136,168,169,170,171].

Voisin et al. exposed mangrove rivulus (*Kryptolebias marmoratus*) for first post-hatching 28 days to 4 and 120 ng/L 17-α-ethinylestradiol, as a model of XE environment [172]. The results showed the effects of 17-α-ethinylestradiol were tissue- and dose-dependent. A total of 31, 51 and 18 proteins were differentially abundant at 4 ng/L in the brain, liver and ovotestis, compared to 20, 25 and 39 proteins at 120 ng/L, respectively. This study demonstrated the long-term effects of early-life endocrine disruption at the proteomic level in diverse estrogen-responsive pathways 5 months after the exposure. The lowest tested and environmentally relevant concentration of 4 ng/L 17-α-ethinylestradiol had the highest impact on the proteome in the brain and liver, highlighting the potency of endocrine disruptors at low concentrations [172].

Hill Jr et al. have researched the concentration-dependent effects of a weak estrogen receptor agonist, 4-NPH and a potent estrogen receptor agonist, 17α-ethinylestradiol (EE) on sex ratios, gonad morphology, vitellogenin (VTG) induction and breeding success in zebrafish (Danio rerio) [173]. Fish were exposed from 2 to 60 days post-hatch (dph) to NPH (10, 30, or 100 microg/L nominal), EE (1, 10, or 100 ng/L nominal), or solvent control (acetone; 0.2% *v*/*v*) in a static-renewal system with replacement every 48 h. The percentage of males at 60 dph changed from 45% in solvent controls to 0% at 10 ng/L EE and 10% at 100 microg/L NPH. In the EE exposure group, a concentration-dependent increase in the number of fish with undeveloped gonads at 60 dph was observed. However, the sex ratios of adults determined at 160 dph revealed no significant departure from 1 male: 1 female, suggesting that exposure of zebrafish to estrogenic chemicals during sexual differentiation and early gametogenesis did not irreversibly alter phenotypic sex. These results suggest that functional reproductive capacity (breeding success) may be more sensitive than gross morphological endpoints (condition, ovo-somatic index, sex ratio) in adult zebrafish exposed to XEs during sexual differentiation and early gametogenesis [173].

Liao et al. have compared the sensitivities of rare minnows during different life stages to 17β-estradiol (E(2)) at environmentally relevant (5, 25, and 100 ng L^−1^ and high (1000 ng L^−1^) concentrations by using VTG and gonad development as biomarkers under semistatic conditions [174]. After 21 days of exposure, VTG concentrations in whole-body homogenates were analyzed. The results indicated that the lowest observed effective concentration for VTG induction was 25 ng L^−1^ E(2) in the adult stage but 100 ng L^−1^ E(2) in the larval and juvenile stages. After exposure in the early life stage, the larval and juvenile fish were transferred to clean water until gonad maturation. No significant difference in VTG induction was found between the exposure and control groups in the adults, but a markedly increased proportion of females and appearance of hermaphrodism in the juvenile-stage group exposed to 25 ng L^−1^ E(2). These results showed that VTG induction in the adult stage is more sensitive than in larval and juvenile stages following exposure to E(2). The juvenile stage may be the critical period of gonad development [174].

## 4. Discussion

On the molecular level, many XEs are known to be structurally related to the steroid hormones produced by the human body (structural similarity to the natural estrogens). Therefore, they have estrogenicity or antiestroginecity and can be bound with the receptors of the organism, manipulating differentiation and modulation of cell proliferation, apoptosis, cytokine production, and cell cycle progression, which should have been controlled over by the endogenous 17β-estradiol [3,6]. On the other hand, some substances, such as Cd, PCB and dioxin, can function as potential endocrine disruptors despite no structural similarity to the natural estrogens. However, the exact mechanism by which this metal Cd may interfere with the reproductive system has not been fully elucidated. The interruption in the steroidogenic pathway by Cd toxic action may be explained in a few different ways [175]. The changes in E2 and progesterone levels may result from the impairment of steroidogenic enzymatic activities by Cd. Under the actions of Cd, conversion of cholesterol to pregnenolone is supposed to be the cause of abnormalities in the metabolism of sex hormones. Thus, endocrine disruptors, including Cd, PCB and dioxin, can exert effects via various pathways other than classical ER-signaling.

Evidence shows the possible impacts of environmental XEs on developing humans and animal groups’ evolution in recent years. The issue could be considered crucial in the long run instead of a short-term (maldevelopment) effect. Some accumulated effects of XEs or EDCs may only occur until the individual matures, reaching adulthood, rather than being effective in a short period [12]. Xenoestrogen-related endometriosis, which damages the reproductive system, precancerous lesions, and proliferation of cancer cells are pertinent examples. For example, cervical cancer or prostate cancer is reported to be relevant environmental estrogens cases. An article reporting how XEs or EDCs affect the female reproductive system, particularly the endometrium, was published by Karoutsou et al. in 2016 [12], in which it includes the idea of the term window. Explaining from a period in a development perspective, the term window of susceptibility means that the developing organisms can be altered by environmental factors, which results in structural, functional, and/or cellular changes. The occurrence of such alterations during these windows could only be identified until the late stages [12].

The use of certain pesticides, which refers to the XEs in relation to the biological damage and adverse events to the reproductive system, was initially documented back in the 20th-century [27,28,29]. Nowadays, the indirect evidence shows that endocrine disruptors are related to various diseases, and their relationship can also be observed in the studies, including metabolic syndrome (especially hypertension and diabetes, obesity), asthma, and various reproductive system-related cancers [6]. Such diseases are not subject, particularly to males or females. Evidence can also be found in breast, cervical, prostate, and vaginal cancer [4].

Previous studies have shown indirect evidence regarding the diseases or harmful organisms triggered by XEs. As mentioned before, these reports focus on the more common XEs, such as DDT and its metabolites DDE, PCBs, DES, 2,3,7,8-TCDD (dioxin), and BPA. As mentioned previously, DDE has a higher affinity and lipophilicity and is not easily catabolized by organisms. Lake Apopka in Florida is seriously affected by DDT and its metabolites. It is discovered that male crocodiles have a micropenis, various abnormalities of the testes, and the cause may be with the exposure of DDT and DDE to crocodiles [108]. Moreover, metallic endocrine disruptors, such as Cd and phytoestrogens, are other examples. Notably, the damages that isoflavones, coumestrol, and clover do to the human body will reflect on the reproductive system and possibly position as the trigger of cancer development. They will also affect other systems, such as the nervous, skeletal, human brains, and even the entire body’s metabolism. However, it can be more beneficial to apply natural and synthetic XEs as they can be considered medications to improve human health, treat and prevent diseases if used appropriately.

The current review has some inherent limitations. The first limitation lies in the focus of the research XEs per se. Because of considering the moral hazard in conducting studies regarding the harmful effects of XEs on humans, many of the studies were observational and animal, thus limiting the application of the review results to humans. Second, studies were conducted with different research designs and subjects (human; animals), and different results were reported with researchers of different skills and training. Therefore, it is somewhat difficult to make comparisons and inferences in the analysis. Moreover, there are fewer randomized controlled trials found on the topic of XEs after selection. Thus, the evidence available in XEs is not as strong as that in other topics. Furthermore, there are many intermediating factors, which affect the outcomes, so the direct causal relationship of XEs effects is hard to be proved. Finally, many of the beneficial and harmful effects of XEs, which have been accumulated in organisms, are dose-dependent and time-dependent, which may not be observed during a shorter period. Maybe a better investigation for the action of XEs can be achieved by a longer and closer follow-up of the research subjects.

Currently, more experiments are required to study the substances in the environment. This is due to the great spectrum of toxins that organisms in the world are exposed to. The worst case of malignant tumors can develop over the years, and many factors also determine the complex process of carcinogenesis development. It is understandable to review the documentary of environmental estrogens and their influences on individual development, as well as to conduct more studies for further clarification.

## 5. Conclusions

As endocrine disruptors, XEs can be either synthetic or natural chemical compounds derived from sources, including diet, pesticides, cosmetics, plastics, plants, industrial byproducts, metals, and medications. By mimicking the chemical structure that is naturally occurring estrogen compounds, even the weakly active compounds could interfere with the hormonal balance with persistency or high concentrations of XEs, thus possibly being associated with the occurrence of the reproductive tract or neuroendocrine disorders and congenital malformations. In contrast, some XEs are not similar to estrogens in structure and can affect the physiologic functions in ways other than ER-ERE ligand routes. In addition to the classical ER-signaling pathway, some endocrine disruptors can exert effects via various pathways other than classical ER-signaling.

The dose-related and time-dependent effects of XEs on organisms should be considered. XEs are most likely to exert tissue-specific and non-genomic actions when estrogen concentrations are relatively low. Current research reported that there is not only a single factor affected by XEs, but opposite directions are also found on several occasions, or even different components stem from the identical endocrine pathway. Thus, the roles of XEs are more challenging and unpredictable in terms of physical health. Although there are numerous studies of XEs or endocrinal disruptors in the literature, many of them are observational and animal, thus limiting the application of the studies to humans. In addition, there are fewer randomized controlled trials of this topic found in the literature. Furthermore, many intermediating factors, which affect the outcomes are difficult to be controlled in these studies, so the direct causal relationship of XEs effects is hard to be proved. Moreover, many of the beneficial and harmful effects of accumulated XEs are dose-dependent and time-dependent, which may not be observed during a shorter period, and may need a longer and closer follow-up of the research subjects. This review provides a current summary of the identification, detection, metabolism, and action of XEs. However, many details of the underlying mechanisms remain unknown and warrant further investigation.

## Figures and Tables

**Table 1:** A summary of in vitro assays for the measurement of estrogenic and antiestrogenic compounds, partially according to Ref [35].

In Vitro Assay	Endpoint of Measurement	Advantages	Limitations	Reference
E-Screen	ERα (+) cell proliferation	Measures physiological endpoint of estrogen action, measures estrogens and antiestrogens	No defined ER expression, no mechanistic data	[33]
Ligand-binding (EDSTAC) ^a^	ERα- or ERβ-binding affinity	Simple, high-throughput method	Does not measure ER activation does not measure physiological response	[36,37]
ER-binding to ERE	ERα- or ERβ-binding affinity to ERE	High-throughput method, various EREs can be used	Does not measure ER activation, low sensitivity, does not measure physiological response	[38,39]
GST pull-down/FRET/ two-hybrid assay	ERα- or ERβ-ligand-dependent association with coactivators	Analysis of molecular interaction, defined ER subtype or ER domain as well as coactivators can be used, measures estrogens and antiestrogens	Does not measure direct ER activation, low throughput, does not measure physiological response	[40,41,42]
Analysis of gene expression	ER-regulated gene expression	Analysis of physiological response, versatile, measures estrogens and antiestrogens	Low throughput	[43,44]
Analysis of enzyme activity	ER-regulated enzyme activity	Analysis of physiological response measures estrogens and antiestrogens	Cell lines or primary cell cultures with active marker enzymes suitable only	[45,46]
Analysis of steroidogenesis (EDSTAC)^a^	Induction and inhibition of estrogen biosynthesis	Analysis of physiological response measures ER-independent pathways	Cells with active steroidogenesis suitable only	[47,48,49,50,51]
The fluorescence-based multianalyte chip platform(a fluorescence-based multiplexed protein microarray)	The proliferative effect in hormone-sensitive cancer cell line MCF-7 was measured with a resazurin assay. Quantification of 10 proteins from MCF-7 cells, representing endpoints of estrogen-and antiestrogen action	High throughput screening. Multiparameter panels, fast and highly specific diagnosis	binding affinities, different concentrations and different periods will be necessary to refine the specific secretion patterns	[52,53,54,55]
Transcriptional activation assays	Luciferase activity	Rapid screening, identification, and characterization of EDCs, the first human ovarian cell bioassay of this kind for detecting estrogens	Response observed in cell lines does not necessarily reflect the toxic or biological potential of a compound in vivo.	[56]
Triple functional small-molecule–protein conjugate mediated optical biosensor	Dye-labeled estradiol (E2) -streptavidin conjugate (estrogenic activity)	Easy-to-use and efficient with the high reusable capability	Expensive	[57]
*Estrogen receptor recombinant yeast* *screening (Y* *ES* *)* *assay*	Estrogenic activity	The simplicity The product of the reporter gene is secreted in the medium, and no cell lysis is required.	The presence of yeast cell wall and active transport mechanisms that may differ from those found in mammalian cells and may affect the activity of some * test * compounds	[58]

^a^ Recommended for the screening of xenoestrogens by EDSTAC/OECD [46,47] ER estrogen receptor; ERE estrogen-responsive element; GST glutathione-S-transferase; FRET fluorescence resonance energy transfer.

## Data Availability

Not applicable.
